# Maternal Hyperhomocysteinemia as a Predictor of Placenta-Mediated Pregnancy Complications: A Two-Year Novel Study

**DOI:** 10.7759/cureus.37461

**Published:** 2023-04-11

**Authors:** Sharmeen I Memon, Neema S Acharya, Sourya Acharya, Jyotsana Potdar, Megha Karnik, Shazia Mohammad

**Affiliations:** 1 Obstetrics and Gynaecology, Jawaharlal Nehru Medical College, Datta Meghe Institute of Higher Education and Research, Wardha, IND; 2 General Medicine, Jawaharlal Nehru Medical College, Datta Meghe Institute of Higher Education and Research, Wardha, IND

**Keywords:** fetal growth restriction, placental abruption, preeclampsia, hyperhomocysteinemia, placenta mediated pregnancy complications

## Abstract

Background

Placenta-mediated pregnancy complications (PMPCs) are a significant contributor to adverse maternal and fetal outcomes. Though the exact cause of the array of pregnancy-related vascular disorders is still unknown, increased maternal serum homocysteine (Hct) levels have been linked to the pathophysiology. Hyperhomocysteinemia (HHct) has been strongly linked with the risk of developing PMPCs such as preeclampsia (PE), fetal growth restriction (FGR), intrauterine fetal death (IUFD), preterm births and placental abruption.

Methodology

The present observational study was carried out on 810 low-risk antenatal women in their early second trimester (13-20 weeks gestation age) in the department of obstetrics and gynecology of a tertiary care rural hospital to identify the significance of abnormally raised maternal serum Hct level in developing PMPCs.

Results

Of the 810 participants studied, 224 (27.65%) had raised Hct levels whereas the rest of the 586 (72.35%) participants had normal Hct levels. The mean Hct level of raised homocysteine group (18.59 ± 2.46 micromol/L) was substantially raised than the normal Hct group (8.64 ± 3.1 micromol/L). It was observed that women with elevated serum Hct levels developed PMPCs significantly more than women with normal serum Hct levels (p-value <0.05). Among HHct subjects, 65.18% developed PE, 34.38% had FGR, 28.13% had a preterm delivery, 4.02% had abruptio placentae and 3.57% had IUFD.

Conclusions

The focus of the current study is on an easy and quick intervention such as assessing the often-ignored levels of Hct during pregnancy that can help predict and prevent PMPCs. It also highlights the necessity for well-thought-out large-scale studies and trials to further examine the phenomena, as pregnancy may be the only time when rural women will have the opportunity to receive advice and to be tested for HHct.

## Introduction

Placenta-mediated pregnancy complications (PMPCs) include preeclampsia (PE), fetal growth restriction (FGR), preterm births, intrauterine fetal death (IUFD), and placental abruption, they affect over 5%-15% of pregnancies and may have a significant adverse effect on maternal and perinatal health. Given that one of the Millennium Development Goals (MDGs) is to decrease maternal, fetal, and neonatal morbidity and mortality, the early detection and treatment of these complications are essential [1].

The exact pathophysiology of PMPCs is not completely known. Their name suggests that the placenta has a major pathophysiologic role. Therefore, it is important to find predictive markers that can detect at-risk pregnancies that can develop PMPCs and their adverse fetomaternal outcomes.

The association between PMPCs and several markers such as low-molecular-weight heparin (LMWH), antiphospholipid antibody (APLA), serum folic acid levels, and serum vitamin B12 levels have been studied in the past, but the findings are still conflicting [2-4]. Recent research has suggested that serum homocysteine (Hct) levels may be linked to several pregnancy-related complications, but the degree to which maternal hyperhomocysteinemia (HHct) increases the risk of PMPCs is yet to be studied in detail [5].

It is been determined that abnormal placental development is associated with elevated maternal serum Hct levels during pregnancy. Evidence shows that the new obstetric risk factor for PMPCs is a rise in the maternal serum Hct level.

Considering the aforementioned context, the current study aims to evaluate the role of maternal Hct level in low-risk antenatal women in their early second trimester in predicting PMPCs and their adverse fetomaternal outcome as an important screening tool in Maternal and Child Health (MCH) care.

## Materials and methods

This observational study was carried out in the Department of Obstetrics and Gynecology of a tertiary care rural hospital over a span of two years from December 2020 to November 2022. The formula used to calculate the sample size is ​​​​​​n = 4pq / (L^2) where p is the expected proportion of PMPCs based on previous studies or the pilot study (15%), q = 100 - p = 85%, L is the allowable error = 20% of p = 3%. The minimum estimated sample size for the present study was 567. A sum of 810 participants was recruited for the study.

Low-risk pregnant women between 13 to 20 weeks of gestational age with spontaneously conceived singleton pregnancies fitting into the given inclusion and exclusion criteria and amenable to follow-up were selected randomly as participants. Women having a history of comorbidities in a previous pregnancy or present pregnancy complicated with cardiovascular, renal, liver, autoimmune disorders, and other associated high-risk factors were excluded. As a routine protocol, all pregnant women were receiving a prophylactic dose of peri-conceptional folic acid (400 micrograms/day).

This study was approved by the Institutional Ethical Committee (IEC) of the Datta Meghe Institute of Medical Sciences (Deemed to be University), approval no. DMIMS(DU)/IEC/2020-21/9340. All the patients gave written consent for enrolling in this study and counselling was done regarding the purpose and method of the study; the patients were informed regarding the need for follow-up till delivery. A predesigned, pretested proforma was used to evaluate the study subjects which included detailed history, clinical examination, and review of investigations in relation to the occurrence of PMPCs later in pregnancy.

Serum Hct level testing

Fasting blood samples of all patients were drawn for serum Hct level testing and collected in a plain vacutainer red bulb. The collected samples underwent centrifugation for about ten minutes at 3000 rpm to extract the serum and were kept at -20°C till use. After re-warming the serum to room temperature, serum Hct quantification was done using Vitros Microtip Hct assay on a VITROS 5600 analyser (Ortho-Clinical Diagnostics, Raritan, New Jersey, USA). The reagent used was HCY 2 Microtip Reagent (VITROS Chemistry Products, Ortho Clinical Diagnostics, Raritan, New Jersey, USA).

Method of Vitros Microtip Hct assay

It is an enzymatic assay based on the enzyme cystathionine beta-synthase. In the assay, tris(2-carboxyethyl) phosphine hydrochloride first reduces the oxidized Hct. Cystathionine beta-synthase is the enzyme that then converts the reduced Hct to cystathionine. Cystathionine beta-lyase then breaks cystathionine to produce ammonia, Hct, and pyruvate. Pyruvate then undergoes reduction by lactate dehydrogenase to lactate, whereby nicotinamide adenine dinucleotide + hydrogen (NADH) is oxidized to nicotinamide adenine dinucleotide (NAD). Oxidized NADH is estimated spectrophotometrically at 340 nm. The amount of NADH is equivalent to the total quantity of Hct in the sample [6].

Hyperhomocysteinemia (HHct)

As there is no defined standard reference range for maternal serum Hct concentrations in early pregnancy, serum Hct level less than 15 micromol/L was taken as normal, and more than 15 micromol/L was considered as HHct on the basis of suggested normal Hct levels as stated in various studies by Choudhury et al. (2020), Chamotra et al. (2020), Nwogu et al. (2020) and Sood et al. (2019) [7-10].

Categorization

Women included in group A were those with elevated serum Hct levels and group B were those with normal serum Hct levels. Both groups were observed prenatally, during labor, and after delivery for the occurrence of any of the PMPCs. Comparison was done between the two groups to determine the significant occurrence of each complication.

Diagnosis of PMPCs

PE, abruptio placenta, FGR, IUFD, and preterm delivery are all PMPCs that are identified as great obstetric syndromes/placental syndromes by the International Federation of Gynecology and Obstetrics (FIGO) in 2019 [11].

**Figure 1:**
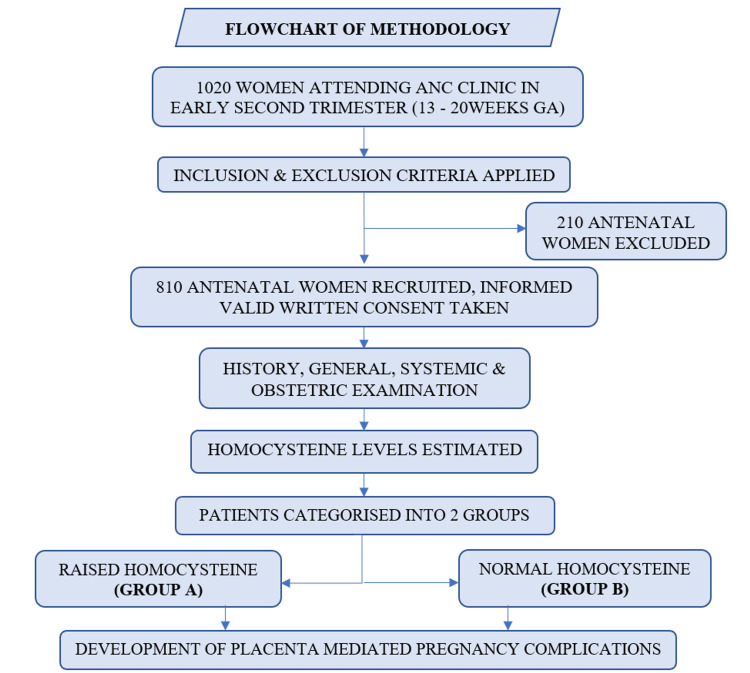
Flowchart showing schematic representation of the study.

Figure 1 shows the schematic representation of the study.

Definition of outcome measures

The criteria for diagnosis of PE includes patients with a blood pressure that was normal earlier and now having a systolic blood pressure of more than equal to 140 mm Hg, or a diastolic blood pressure of 90 mm Hg or more, recorded twice within a period of four hours, after the gestational age of 20 weeks with proteinuria of more than 300 mg per 24-hour, a protein/creatinine ratio of more than 0.3 mg/dL, or dipstick reading of two plus or more, or newly developed hypertension along with any of the following conditions: thrombocytopenia (platelet count less than 1,00,000/mm3), renal insufficiency, pulmonary edema, signs of liver function impairment, and a newly developing headache that is not improving with medical care [12].

The early placental separation from the lining of the uterus before the end of the second stage of labor is defined as placental abruption or abruptio placentae [13].

Neonates with a weight less than the 10th percentile for their gestational age are considered as small-for-gestational-age. Fetal growth restriction is a term frequently used to describe neonates who have low birth weights and are small for gestational age [14].

Preterm birth is any birth that occurs at less than 37 weeks of gestational age or at less than 259 days since the last regular menstruation of the woman [15].

In accordance with World Health Organization (WHO)/ International Classification of Diseases (ICD) guidelines, IUFD is when a fetus dies after reaching a birth weight of 500 g or, in the absence of a birth weight estimate, after 22 weeks of gestation or a 25 cm crown-to-heel measurement [16].

Statistical analysis

The data were recorded continuously in a predefined, predetermined tool and transferred to a spreadsheet (Microsoft Excel). Data were then analyzed for comparison between the two groups using appropriate statistical methods. By using the chi-square test, descriptive and inferential statistics were done for the statistical study. The following metrics were assessed that includes sensitivity, specificity, positive predictive value, and negative predictive value. Software SPSS 17.0 (SPSS Inc., Chicago, IL, USA) and Graphpad Prism 7.0 (Graphpad Software, La Jolla, CA, USA) were used for the analysis, and a p-value of less than 0.05 was considered significant.

## Results

As mentioned in Table 1, 810 low-risk antenatal women in their early second trimester (13-20 weeks gestation age) were recruited, out of which 224 (27.65%) women had raised serum Hct level (≥ 15 micromol/L; group A) and 586 (72.35%) women had normal serum Hct level (< 15 micromol/L; group B). They were followed for maternal and fetal outcomes throughout pregnancy till delivery.

**Table 1:** Mean serum homocysteine level (micromol/L) amongst group A and group B.

Total population (n=810)	Raised Homocysteine ( ≥ 15 micromol/L) GROUP A	Normal Homocysteine ( < 15 micromol/L) GROUP B
810	224	586
100%	27.65%	72.35%

The mean serum Hct level in women who had normal Hct level was 8.64 ± 3.1 micromol/L while it was 18.59 ± 2.46 micromol/L for those who had raised Hct level. The mean age of raised and normal Hct level groups was 26.54 ± 4.13 and 25.99 ± 4.06 years respectively, with a majority of women in the 20-30 years age group. Maximum number of patients in both the groups belonged to rural areas i.e., 177 (79.02%) patients in group A and 464 (79.18%) patients in group B. A majority of women in the raised Hct level group (57.59%) and in the normal Hct level group (55.29%) belonged to the lower socioeconomic class as per the modified Kuppuswamy scale. It was found that 47.7% of patients in group A and 43.52% of patients in group B were primigravidas and 52.23% of patients in group A and 56.48% of patients in group B were multigravida. However, with regard to sociodemographic parameters, the two groups were comparable because there was no significant statistical difference between them. The distribution of different sociodemographic parameters among the two groups is presented in Table 2.

**Table 2:** Sociodemographic distribution of patients amongst group A and group B. S: Significant, NS: Not significant

Parameter	Sub Category	Raised Homocysteine Group A (n=224)	Normal Homocysteine Group B (n=586)	p-value
Serum Homocysteine (micromol/L)	Mean ± SD	18.59 ± 2.46	8.64 ± 3.1	p < 0.001^S^
Age (years)	<20	6 (2.68%)	14 (2.39%)	p=0.998^NS^
	20-24	81 (36.16%)	212 (36.18%)	
	25-29	84 (37.5%)	224 (38.23%)	
	30-34	45 (20.09%)	117 (19.97%)	
	35-39	8 (3.57%)	19 (3.24%)	
	Mean ± SD (Range)	26.19 ± 4.19 (19 - 36)	26.23 ± 4.09 (19 - 37)	p=0.894^NS^
Area of Residence	Rural	177 (79.02%)	464 (79.18%)	p=0.959^NS^
	Urban	47 (20.98%)	122 (20.82%)	
Socio economic Status	Lower class	129 (57.59%)	324 (55.29%)	p=0.679^NS^
	Upper lower class	7 (3.13%)	26 (4.44%)	
	Lower middle class	64 (28.57%)	165 (28.16%)	
	Upper middle class	8 (3.57%)	33 (5.63%)	
	Upper class	16 (7.14%)	38 (6.48%)	
Parity	Multigravida	117 (52.23%)	331 (56.48%)	p=0.276^NS^
	Primigravida	107 (47.77%)	255 (43.52%)	

As mentioned in Table 3, it was observed that of the total 27.65% (n = 224) of the pregnancies with increased Hct, 28.13% (n = 63) had preterm deliveries which were significant statistically (p < 0.001). PE which occurred in 65.18% (n = 146) of the pregnancies with elevated Hct (p 0.001) also showed statistical significance; 34.38% (n = 77) of the pregnancies with elevated Hct was significantly associated with FGR (p < 0.001). Placental abruption was shown to occur in 4.02% of subjects with Hct levels greater than 15 micromol/L and 0.85% of subjects with Hct levels less than 15 micromol/L. This difference was found to be statistically significant (p = 0.002). When compared to individuals with normal Hct levels (0.85%), those with significantly increased Hct had a considerably greater percentage (3.57%) of IUFD.

**Table 3:** Distribution of patients according to PMPCs amongst group A and group B. S: significant, PMPCs: placenta-mediated pregnancy complication, FGR: fetal growth restriction, IUFD: intrauterine fetal death.

PMPCs	Raised Homocysteine GROUP A (n=224)	Normal Homocysteine GROUP B (n=586)	TOTAL	Chi square value	p value
Preeclampsia	146 (65.18%)	82 (13.99%)	228 (28.15%)	209.92	p < 0.001^S^
Abruptio placentae	9 (4.02%)	5 (0.85%)	14 (1.73%)	9.55	p = 0.002^S^
FGR	77 (34.38%)	14 (2.39%)	91 (11.23%)	166.25	p < 0.001^S^
Preterm Births	63 (28.13%)	21 (3.58%)	84 (10.37%)	105	p < 0.001^S^
IUFD	8 (3.57%)	5 (0.85%)	13 (1.6%)	7.58	p < 0.001^S^

As depicted in Table 4, there were statistically significant differences in the occurrence of PMPCs between the two study groups (p-value <0.05); 91.07% of patients with raised Hct levels developed PMPCs in comparison to 14% of patients with normal Hct levels.

**Table 4:** Distribution of total number of patients developing PMPCs amongst group A and group B. TP: true positive, TN: true negative, FP: false positive, FN: false negative, PMPCs: placenta-mediated pregnancy complications

PMPCs	Raised Homocysteine GROUP A (n=224)	Normal Homocysteine GROUP B (n=586)	TOTAL
Present	204 (TP) (91.07%)	82 (FP) (14%)	286 (35.3%)
Absent	20 (FN) (8.93%)	504 (TN) (86%)	524 (64.7%)
TOTAL	224 (100%)	586 (100%)	810 (100%)

Table 5 depicts that the sensitivity, specificity, positive predictive value, and negative predictive value of Hct as a predictor of PMPCs were 91.07%, 86%, 41.97%, and 98.86%, respectively.

**Table 5:** Distribution of patients according to performance of maternal serum homocysteine levels as a predictor of placenta-mediated pregnancy complications (PMPCs).

Performance of homocysteine as a predictor of placenta mediated pregnancy complications (PMPCS)	Percentage
Sensitivity	91.07%
Specificity	86%
Positive Predictive Value	41.97%
Negative Predictive Value	98.86%

Most of the present literature provides evidence to prove that raised Hct levels are indicative of PMPCs and maternal and neonatal adverse risks. However, further studies in larger sample sizes are required to use serum Hct levels as a standard investigation in early pregnancy to predict the development of PMPCs and adverse maternal, fetal, and neonatal outcomes.

## Discussion

PMPCs are a compilation of disorders that have been studied extensively yet not fully understood and continue to pose a major threat to fetomaternal health during pregnancy. Although PMPCs usually present as a new onset disease in the third trimester, several studies have shown that the underlying mechanism behind it begins early in pregnancy and hence it becomes logical to search for predictive tools before its onset [17]. Many predictive markers for the disease have been studied over the years but none have proved to be a gold standard. This study was carried out with a rationale to evaluate a non-invasive marker like serum Hct level and study its efficacy to predict PMPCs.

Placenta plays a major role in the pathophysiology of raised serum homocysteine levels causing the development of various PMPCs. HHct results in the production of superoxide free radicals and hydrogen peroxide which results in endothelial damage and constriction of blood vessels in villi, decreased blood flow at the maternal-fetal interface, and eventually poor maternal and newborn outcomes by oxidatively harming the endothelial cells. Furthermore, HHct stimulates cell death which results in trophoblast dysfunction. Placental perfusion is impacted by the reduction of nitric oxide that endothelial cells release, as well as by induction of platelet buildup and promotion of thrombosis, which activates a coagulation pathway, eventually leading to endothelial injury [17].

In the present study, as mentioned in Table 1, HHct was recorded in 27.65% of low-risk antenatal women in their early second trimester which is in concordance with the study by Oluwole et al. (2020) where HHct was reported in 24.6% of antenatal patients [18]. Also, Bergen et al. (2012) in a study reported HHct in 22.2% of pregnant women [19].

A statistically significant difference was found between the two groups with respect to mean serum Hct levels (p < 0.001). This was supported by the research carried out by Chamotra et al. (2020), where the mean Hct level in the increased and normal Hct groups were 23.26 ± 10.77 micromol/L and 8.99 ± 2.47 micromol/L respectively with statistically significant difference (p < 0.001) [8].

The two groups were comparable with regard to sociodemographic characteristics as seen in Table 2. In terms of age, socioeconomic status, place of residence, and parity, women who had raised Hct concentrations did not differ substantially from those who had normal Hct concentrations which is in concordance with studies by Nwogu et al. (2020), Oluwole et al. (2020) [9,18]. However, in a cohort study undertaken by Chaudhry et al. (2019), a significant correlation between socioeconomic status and the severity of PMPCs was found [20].

The present study results with respect to a significant development of PE in raised Hct level group as shown in Table 3 were identical to the study done by Maru et al. in 2016, who discovered a significant association of high Hct levels with the onset of PE [21]. Raised serum Hct concentration was identified to be a high risk for the development of PE by Serrano et al. (2018) in another case-control research [22]. However, in research done by Oluwole et al. in 2020, no association was found between elevated Hct and PE [18].

Again, in line with this study, Klai et al. (2011) observed a similar positive association between high Hct level and prediction of placental abruption [23]. In a retrospective study by Budde et al. (2007), it was found that increased Hct expression is a high-risk factor for the occurrence of placental abruption, contrary to a study by Chaudhry et al. (2019) who concluded that increased serum Hct concentration has no association with placental abruption [20,24].

A study by Gaiday et al. (2022) on 315 singleton pregnant women reported that patients with FGR had a high concentration of Hct between 10 to 14 weeks of gestational age. According to the findings of this study, measuring the serum Hct concentration in early pregnancy can be used to predict the occurrence of FGR [25]. The current study also states a significant association of raised serum Hct level with the occurrence of FGR as seen in Table 3. Contrary to the current study, Infante-Rivard et al. (2003) in his study reported that increased Hct decreases the likelihood that a woman would deliver a baby with FGR [26].

Elevated maternal serum Hct levels could be considered as a biomarker to predict preterm delivery, according to the findings of the current study. This is in accordance with another study by Nwogu et al. (2020) which involved 200 participants with singleton pregnancies that were at or before 14 weeks of gestation and found that when compared to antenatal women with normal Hct levels, the incidence of preterm delivery was seven times higher in the HHct group [9]. Contrary to a study by Mascarenhas et al. (2014), wherein he found no statistically significant association between Hct levels and preterm births (p - 0.8348) [27].

In the current study as depicted in Table 3, a significant statistical difference (p-value=0.006) was discovered between the two study groups in terms of the occurrence of IUFD. Maru et al. obtained similar results in a study conducted in 2016, which included 22 patients with IUFD, 20 of whom had HHct and two of whom had normal Hct levels [21]. However, research by Nwogu et al. in 2020 discovered no correlation between increased Hct level and pregnancy loss (p-value=0.118) [9].

In the present study, the total PMPCs among the groups with raised Hct level was 204 (91.07%) patients, and among the group with normal Hct level was 82 (14%) patients as mentioned in Table 4. This is consistent with a study by Chaudhry et al. (2019) which found that a greater plasma Hct level was strongly linked to an elevated risk of any PMPCs (p-value=0.0007) [20].

The present study, as mentioned in Table 5, represents the performance of serum Hct level as a screening tool in the detection of PMPC. It is depicted by the sensitivity of 91.07%, specificity of 86%, positive predictive value of 41.97%, and negative predictive value of 98.86%.

Table 6 summarizes the conclusions of various studies related to the present study showing an association between HHct and the occurrence of various PMPCs.

**Table 6:** Studies showing an association between hyperhomocysteinemia and the occurrence of various PMPCs. PMPC: placenta-mediated pregnancy complication, PE: preeclampsia, FGR: fetal growth restriction, AP: abruptio placentae, IUFD: intrauterine fetal death, Hct:: homocysteine, HHct: hyperhomocysteinemia

Author	Year	PMPCs studied	Conclusion
Present Study	2023	All PMPCs	Serum Hct level can be used as a predictor of PMPCs
Gaiday et al. [25]	2022	FGR	Increased serum Hct levels may be used to predict FGR.
Oluwole et al. [18]	2020	PE	No significant association between maternal HHct and preeclampsia.
Chamotra et al. [8]	2020	AP, FGR	HHct is linked to adverse fetomaternal outcomes involving AP and FGR.
Nwogu et al. [9]	2020	Preterm birth	Significant association observed between maternal HHct and preterm birth.
Serrano et al. [22]	2018	PE	Raised Hct was one of the major risk factors for development of PE.
Qiu et al. [28]	2017	Preterm birth	Elevated Hct levels in the maternal serum may be used as a biomarker to indicate preterm birth.
Maru et al. [21]	2016	PE, AP, IUFD	Hct is a reliable predictive marker for PE, AP, IUFD and other adverse fetomaternal outcomes.
Dhobale et al. [29]	2012	Preterm birth	Women delivering preterm have higher homocysteine concentrations.
Ghike et al. [30]	2013	PE	The severity of PE and serum Hct levels may be strongly correlated.
Klai et al. [23]	2011	PE, AP, FGR	Increased Hct levels predict the development of PE, AP and FGR.
Infante Rivard et al. [26]	2003	FGR	Mothers with lower homocysteine concentrations give birth to FGR babies.

Limitations

Further studies in larger sample sizes are required to use maternal serum Hct concentrations as the standard to predict PMPCs in early pregnancy.

Recommendations

Periconceptional dietary counselling regarding having a diet rich in folate and vitamin B complex and additional supplementation with a high dose of folic acid, vitamin B12, and vitamin B6 daily can help in significantly reducing the Hct levels and prevent the development of PMPCs.

Since it was observed in the present study that serum Hct level testing had a strong negative predictive value, it can be beneficial in identifying those patients who do not need vigorous fetomaternal monitoring and the resources can be reserved only for those low-risk patients who have HHct evaluated in the early second trimester of pregnancy.

## Conclusions

Results of the present study conclude that the occurrence of PMPCs was significantly higher in women who had raised serum Hct levels than those women who had normal serum Hct levels. Thus, maternal HHct can be used as a predictor of the development of PMPCs and suggests that serum Hct level can be used to screen low-risk antenatal patients in the early second trimester.
